# Finding the right candidate: Developing hiring guidelines for screening applicants for clinical research coordinator positions

**DOI:** 10.1017/cts.2021.853

**Published:** 2021-09-22

**Authors:** Elaine Fisher, Rebecca S. Thomas, Melinda K. Higgins, Charlie J. Williams, Ikseon Choi, Linda A. McCauley

**Affiliations:** 1 Emory University, Atlanta, GA, USA; 2 Emory Healthcare, Atlanta, GA, USA; 3 University of Georgia, Athens, GA, USA

**Keywords:** Human resource recruiter, clinical research coordinator (CRC), clinical research, hiring process, principal investigator

## Abstract

**Impact:**

The success of any clinical research team is dependent on hiring individuals with the experience and skill set needed for a specific research project. Strategies to improve the ability of human resource (HR) recruiters to screen and advance qualified candidates for a project will result in improved initiation and execution of the project.

**Objective/Goals:**

HR recruiters play a critical role in matching research applicants to the posted job descriptions and presenting a list of top candidates to the PI/hiring manager for interview and hiring consideration.

**Methods/Study Population:**

Creating guidelines to screen for applicant qualification based on resumes when clinical research positions have multiple levels of expertise required is a complex process of discovery, moving from subjective rationale for rating individual resumes to a more structured less biased evaluation process. To improve the hiring process of the research workforce, we successfully developed guidelines for categorizing research coordinator applications by level from beginner to advanced.

**Results/Anticipated Results:**

Through guideline development, we provide a framework to reduce bias and improve the matching of applicant resumes to job levels for improved selection of top candidates to advance for interviewing. Improved applicant to job matching offers an advantage to reduce hiring time, anticipate training needs, and shorten the timeline to active project engagement. These guidelines can form the basis for initial screening and ultimately matching individual qualities to project-specific needs.

## Introduction

Clinical research coordinators (CRCs) are responsible for overseeing the day-to-day operations of clinical research trials and studies. Recruiting and hiring a qualified individual to coordinate research studies can be the key to the successful launch and execution of many research projects. There are currently an estimated 56,700 CRCs in the USA with the job market expected to grow by 9.9% between 2016 and 2026. Projecting over the next 10 years, the estimation is that the USA will need 11,200 CRCs, 5,600 additional CRCs plus the retirement of 5,600 existing CRCs [1].

Responsibilities of a CRC vary widely depending on the type of study; number and expertise of current team members; expectations of the principal investigator (PI); and experiences, skills, and competencies a new CRC brings to the job. There is no “standard” research coordinator job; therefore, this unique nature of clinical research trials and studies can make the matching of candidates to coordinator positions challenging. Replacing a coordinator who cannot execute the job responsibilities can be a nightmare for a PI and result in a delay of the study execution. A key step in the hiring process is working with human resource (HR) recruiters to identify top candidates to interview. Fifty-two percent of talent acquisition leaders report the hardest part of recruitment is identifying the “right candidates” from a large and diverse applicant pool [2]. Too often PIs review and reject resumes of proposed applicants from the HR recruiter, sending the HR recruiter back to the applicant pool to provide additional candidates for consideration. It is particularly challenging when selecting top candidates for multi-leveled jobs. This requires screening candidate resumes for specific skills, project roles/responsibilities, and total years of experience; and for entry-level positions, being able to identify important transferrable skills to match job requirements.

The competencies needed in CRC roles are broad, ranging from a global understanding of research processes, experience meeting specific regulatory, and reporting requirements, to clerical or supervisory activities. Most organizations provide standard job descriptions that are globally written and open to interpretation by both the recruiter and applicant. Overly broad job descriptions prevent the accurate matching of candidates to specific needs of a research study. As a result, applicants may have little understanding of the position requirements and distinctions between entry-level, intermediate, and advanced positions and may apply for positions requiring a wide range of expertise, hoping the HR recruiter will be able to identify which components of their academic preparation, experiences, and skill set provide a “best fit” to earn an interview for a position.

The burden then falls on the HR recruiter to filter through often hundreds of resumes for a single CRC position to select top applicants for consideration. It is also common for HR recruiters in large academic health centers to be reviewing resumes for 50 or more diverse jobs at a time. If required skills and competencies particularly for entry-level positions are unspecified or unclear, the HR recruiter may overlook top candidates or send forward unqualified candidates. This is an inefficient use of time for the recruiter, PI, and applicant and results in hiring delays. For research-related positions, especially on federally funded grants, these inefficiencies can lead to missed project milestones.

Many PIs may be hiring research personnel for the first time and have a limited understanding of what skills and competencies are needed during the study life cycle. They may not be able to match the salary resources on the project with the competencies they desire in a research coordinator. A posting will be for an entry-level position (using the salary available) with job expectations only seen in more advanced candidates.

Resumes from job applicants may be written very broadly with little specificity on competencies of the individuals including skills that could be transferable from other non-research coordinator positions. HR recruiters are essential to the hiring process both in developing specific job descriptions and in conducting initial resume screening to judge which resumes are good matches for specific levels of research coordinator positions. Given the important responsibilities of HR recruiters in the hiring of CRCs, we conducted a project focused on improving the process of successfully recruiting candidates for research coordinator positions. The project had two goals 1) examine current HR hiring practices in a large research-intensive, academic-medical center; and 2) to develop CRC hiring guidelines for use by HR recruiters to improve the matching of top candidates to project and PI needs. This paper describes how we used a mixed method approach to understanding the most common practices for hiring CRCs and the process of developing a more streamlined process of screening and hiring CRCs for clinical research positions.

## Common HR Hiring Practices

For the qualitative, exploratory phase of this project, we conducted 30-45 minute interviews with HR administration (*n* = 3) and HR recruitment specialists (*n* = 4) to better understand the process currently used to match CRC resumes to posted job opportunities and how candidates are advanced for consideration to PIs and potential hire. We supplemented the descriptions of their work processes with quantitative data on the volume of positions and numbers of applicant resumes HR recruiters typically screen.

### Job Postings

Ideally, the PI/hiring manager submits a clearly written job description and has a direct phone conversation with the HR recruiter prior to posting the job. One HR recruiter pointed out the need to “handhold” PIs/hiring managers, often calling the PI/hiring manager to request more specific information about the job description or for assistance with screening parameters. The HR recruiter stated, “they [PI/hiring manager] usually never return my phone call.”

### HR Resume Screening

Once the job is posted, applicants submit their resumes through the applicant tracking system. HR interviewees were quick to describe the laborious procedures in screening CRC resumes. One administrator remarked, “One of the chief points of pain is the front-end volume issue. This limits the HR recruiters” ability to be sourcing quality candidates rather than filtering through 300 resumes to find 30 qualified candidates.”

### HR Work Volume

Between May 2019 and August 2020, our academic health center received 20,622 applicant resumes for 201 CRC job postings. The average (mean ± SD) number of applications for each CRC level posting was CRC 1, 176 ± 98; CRC 2, 117 ± 52; CRC 3, 99 ± 47; and CRC 4, 76 ± 29. The range of applications for each job opportunity ranged from 1–595 indicating a large interest from individuals seeking positions as CRCs.

The HR recruiter, typically weekly, does a first-pass for applications/resumes not meeting minimum job requirements. These applications are removed from the pool without further review. Current practice for screening the remaining resumes involves reviewing each application using traditional information retrieval techniques, that is, Boolean retrieval methods, searching open texts for key search criteria. The HR recruiter next selects 5–10 top candidates and submits the list of candidate resumes to the PI/hiring manager for review.

### Assisting the PI/Hiring Manager in Candidate Selection

At the point where candidates are put forward to the PI/hiring manager for consideration, a call may come to the HR recruiter from the PI stating the candidates do not meet their needs. A repeated comment echoed by HR recruiters from conversations with the PI is the statement made by the PI, “I”ll know it [the correct candidate for the job] when I see it.” This sends the HR recruiter back to the candidate pool to select additional candidates for hiring consideration and/or the PI/hiring manager asking to see all resumes and them proceeding to independently screen candidates. Table 1 displays descriptive statistics on filling positions (days) by CRC level. Time-to-fill is defined as time of job posting to the day of candidate offer and acceptance.

**Table 1.** Time-to-fill (days) a clinical research coordinator (CRC) position by CRC level (*n* = 178)

	CRC 1 (*n* = 74)	CRC 2 (*n* = 78)	CRC 3 (*n* = 20)	CRC 4 (*n* = 6)
Mean ± SD	55 ± 45	56 ± 45	75 ± 66	68 ± 36
Median	44	46	48	57
Range (days)	4–222	1–292	14–259	36–128

**Fig. 1.**
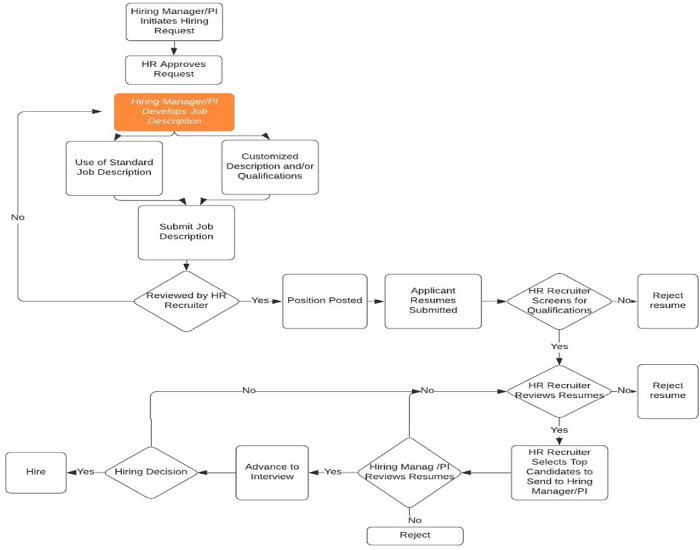
Hiring process flowchart.

Based on our interviews, the following HR Hiring Flow Chart, Fig. 1, displays the laborious steps used to screen resumes and reach the goal to hire.

The high volume of applications for CRC positions and the lengthy process of recruiting, screening, and hiring CRCs is inefficient giving the substantial knowledge that is known about clinical research competencies. This process can also be extremely frustrating to PIs wanting to quickly launch a funded research project. Given these complexities, the second phase of the project focused on the development of resume” screening guidelines based on applicant qualifications and experiences to ultimately improve the hiring process of CRCs.

## Materials and Methods

### Guideline Development for Screening CRC Qualifications by Job Level

Using a retrospective approach, we obtained electronic records of resumes submitted over a 12-month period to the Human Resources Department of a large academic research-intensive institution. Between April 2018–19, 20,095 resumes were received for 225 advertised CRC positions.

Most of the applicants (90%) applied for an entry-level positions, CRC 1 (56%) or CRC 2 (34%). The majority of applicants applied to multiple positions and/or levels of positions resulting in 8032 unique individuals. For the purpose of our analysis, we reduced the sample to include only one position per applicant (5741), with the unique applicant resume included in the CRC level for the highest level of position to which they applied. Table 2 displays how the total number of applicant resumes was reduced to the final analytical sample.

**Table 2.** Initial and final resumes by clinical research coordinator (CRC) level

*20,095 total resumes*			
CRC I	CRC II	CRC III	CRC IV
11,276	6777	1801	241
*5741 final unique resumes*			
CRC I	CRC II	CRC III	CRC IV
2733	1947	837	224

A stratified sampling methodology was used to sort resumes into analytical batches of 50 resumes. Batches of 50 resumes were randomly selected from each CRC level in the proportion represented in the final unique resume pool. Thus, in each batch of 50 resumes, we included 23 CRC 1 resumes (46%), 17 CRC 2 resumes (34%), 8 CRC 3 resumes (16%), and 2 CRC 4 resumes (4%).

Two experts familiar with conducting clinical research studies and having an understanding of the skills, competencies, and possible transferrable skills appropriate for hiring to different levels for CRC positions, independently reviewed the batches of 50 stratified resumes. Blind to the level of CRC position to which the applicant applied, each reviewer provided a rating for what level of CRC position best matched the qualifications on the resume. Reviewers then met to adjudicate ratings with the final determination made by consensus. In the review process, the reviewers developed consensus on the traits associated with each level of CRC position. During the consensus process, guideline criteria evolved for assigning resumes to a level of CRC.

The process of resume evaluation and adjudication continued until moderate–good interrater agreement was achieved as determined using Fleiss Kappa [3,4].

### Process of Developing Hiring Guidelines

Adjudication of the ratings by reviewers highlighted the need to clarify the types of academic preparation and employment experiences by CRC level, particularly at the entry level (CRC 1 & CRC 2) where no or little evidence of experience in a clinical research field was noted on the resume. Two questions emerged, 1) What constitutes a transferrable skill for candidates with no previous research experience?; and 2) When is an applicant considered “not qualified?”

For entry-level positions, two areas of transferrable skills were considered essential by reviewers, 1) academic preparation in a healthcare or scientific field; and 2) clinical experience either in a direct or indirect patient care role in a clinical setting. Academic preparation at the CRC 1 level was defined by a certificate, diploma, associate degree, or bachelor’s degree level so long as there was a focus in a scientific or health-related field. Candidates were considered “not qualified” if resumes noted only work in non-healthcare, customer-facing roles, that is, waiter, receptionist, or general office work. Table 3 shows examples jobs in healthcare, science, or clinical settings that could include skills transferable to a CRC entry-level position.

**Table 3.** Transferrable skills: clinical settings, clinical roles, and exclusions

Clinical setting	Clinical role
Hospital Clinic Healthcare Provider Office Healthcare Facility	Patient Service(s) Coordinator, Patient Care Coordinator, Clinical Coordinator, Unit Secretary Clinical Service Representative Medical Scribe, Medical Secretary Pharmacy Technician Patient Service(s) Coordinator, Patient Care Coordinator, Clinical Coordinator, Unit Secretary Clinical Service Representative Medical Scribe, Medical Secretary, Pharmacy Technician Phlebotomist Tumor Registrar Pre-/Post-Award Administrator Internship in a scientific or health-related area (1000 documented hours)

Exclusions: Business Analyst, Financial Navigator, Massage Therapist.

### Assessment of Prior Clinical and Research Experience

All CRC positions beyond entry level were required to have some prior clinical or laboratory research experience. Laboratory or bench researchers were required to have a greater number of years of experience in research to qualify for higher CRC level jobs. While laboratory workers were viewed as having overall knowledge of the research process, lack of patient contact and experiences with basic CRC functions, for example, screening and informed consent, patient scheduling, adverse events reporting, resulted in assigning applicants to lower CRC levels.

For applicants with a doctoral degree or training as a foreign-trained doctor, further considerations were made based on evidence of having clinical research experience beyond academic preparation. For the top position, CRC 4, expertise was defined by years of clinical research experience and having attained a recognized clinical research-based certification. Certification by research-based organizations typically requires clinical research experience of 2000–3000 hours or approximately 1 to 1 ½ years of full-time work.

### Analysis of Reviewer Agreement

The goal of the review of resumes by experts in clinical research was to develop consensus guidelines that could be used by HR screeners. Initial reviewer ratings were compared to the final adjudicated rating in order to determine those qualifications that had the largest range of non-agreement. Rater agreement was also determined by computing Fleiss Kappa, which assesses the interrater agreement as a measure of reliability among the various raters [3]. If raters are in complete agreement then Kappa will equal 1. If there is no agreement among reviewers, Kappa will equal 0. The relative “effect size” of the reported Kappa values is also subjectively described using ratings provided by Altman [5]: strength of agreement <0–0.20 poor, 0.21–0.40 fair, 0.41–0.60 moderate, 0.61–0.80 good, to 0.81–1.00 very good agreement.[5] The correlation between guideline revision sequence and Kappa for that batch was computed using Spearman’s rho, which is appropriate for the small number of batches and ordinal sequence. All analyses were performed using IBM SPSS v.26 (IBM, 2019).

## Results

The final dataset included a review of 300 resumes rated over six (6) batches of 50 resumes each. Of the total analyzed resumes, 14% (42) were rated as not qualified, 39% (117) as CRC 1; 23% (69) as CRC 2; 21% (64) as CRC 3; and 3% (8) as CRC 4. Over 70% of applicants applied for jobs did not match with their qualifications. Table 4 displays the results of the reviews and adjudications that occurred. In the initial review of Batch 1 with no guidelines, there was little agreement among the reviewers on if candidates were qualified for positions. This lack of agreement led to discussions on transferrable skills, level of education as scientific, health-related, and nonscientific, non-health-related degrees, and required years of clinical and research experience. Agreement on these guidelines led to subsequent improvement and consensus on identifying not-qualitied applicants. Ratings of applicants for level 2 CRC positions showed the most variability, determined in a large part bt the inability to accurately calculate the exact months/years of experience held by the applicant. Rating agreement improved with the determination of an agreed method to calculate months of experience. For applicants with multiple jobs, discrepancies occurred in totaling years of experience based on variations in job titles, limited details of roles and responsibilities, and/or clarity of time in each position.

**Table 4.** Guideline evolution: overall agreement (Kappa) by guideline sequence

Guideline version	Batch	Kappa (p-value)	Guideline revision based on adjudication
0 (none)	1A	0.161* (*p* = 0.045)	Distinguished between a qualified/not-qualified applicant resumes Defined transferrable skills Defined key terms: clinical role/setting, clinical experience, clinical research experience Set requirements for clinical experience and academic preparation by level
1	2	0.453 (*p* < 0.001)	Revised years of clinical experience by level Categorized academic degree as scientific/health-related field or nonscientific/non-health-related field Refined clinical research coordinator (CRC) 2, 3, 4-degree requirements, years of clinical research, and clinical experience requirements Required clinical research certification for a CRC 4
2	3	0.407 (*p* < 0.001)	Updated glossary examples of clinical settings, clinical experience by job titles, and clinical research experience Revised years of experience in clinical roles and years of clinical research experience Developed categorization of foreign-trained MD and bench (laboratory) research personnel
3	4	0.555 (*p* < 0.001)	Removed clinical role/setting experience requirement from CRC 2–4 and required a minimum of 1-year clinical research experience for those with a scientific degree Revised laboratory (bench) scientist requirements Removed academic experience as a research assistant unless it involved over 1000 contact hours. Rationale: academic experience was typically data entry or participation in a component of the research project but not full engagement in a project
4	5	0.608 (*p* <0.001)	Increased the number of years required for a laboratory (bench) researcher
5	1B	0.593 (*p* < 0.001)	Final guidelines

* Kappa computed for Raters 1 and 2 for Guidelines 0 (Batch 1A), Kappa computed for 3-Raters for remainder of development.

Discrepancies among reviewers for higher-level positions occurred initially when developing level requirements for PhD, foreign-trained doctors, and laboratory researchers. Rating agreement improved with the establishment of clear guidelines for evaluating the types of experience of these individuals. Additionally, the limited number of applicants for CRC 3 and CRC 4 jobs in the pool influenced lower agreement for levels CRC 3 and CRC 4. Guidelines were revised over the series of batches reviewed. Key guideline modifications were made during adjudication and overall agreement by guideline sequence improved over time (Table 4).

Table 5 shows that based on the final hiring guidelines that evolved from the process, good to very good agreement was achieved among raters for the CRC levels of not qualified [NQ], CRC 1, CRC 3, and CRC 4. Fair agreement was noted for level CRC 2. Inability to accurately calculate exact employment dates/experience led to the lower agreement for CRC 2.

**Table 5.** Rater agreement by clinical research coordinator (CRC) level of batch 1 using final guidelines

CRC Level	Kappa (κ)	Standard error	Z	*p*-value	95% CI lower bound	95% CI upper bound
Not qualified	0.742	0.082	8.999	<0.001	0.737	0.747
CRC 1	0.604	0.082	7.319	<0.001	0.598	0.609
CRC 2	0.396	0.082	4.796	<0.001	0.390	0.401
CRC 3	0.530	0.082	6.432	<0.001	0.525	0.536
CRC 4	1.000	0.082	12.124	<0.001	0.995	1.005

CI = confidence interval.

## Discussion

The success of any research enterprise is dependent on the ability to recruit and screen qualified individuals who can meet the project needs and competencies expected. The skills needed to execute increasing complex study designs are increasing, and while there appears to be robust interest in careers in research, matching individuals and their qualifications to specific project needs can be a challenge [6]. The recruitment and employment of CRCs is a multi-step process, with the HR recruiter often the invisible partner in the initiation of a successful hiring process. While much work has been done on research competencies and tasks associated with CRC positions, HR recruiters may not be highly familiar with these competencies. Given the large number of applications for research positions, HR professionals need structured guidelines for screening potential candidates to ultimately improve and accelerate the hiring process. Creating guidelines can be a complex process of discovery, moving from subjective rationale for rating individual resumes to a more structured, less biased evaluation. Decisions based on subjective rationale can carry implicit bias, revealing attitudes and stereotypes about the unconscious manner in which decisions were made when reviewing resumes. In this project, by using a consensus strategy, implicit biases became explicit, highlighting beliefs that may lead to bias in candidate selection. For example, an international candidate who makes several errors in grammar and punctuation, despite having the requisite skills, competencies, and years of working in the field of clinical research, could be eliminated from consideration based on resume appearance. The iterative process of this project resulted in a more conscience awareness of the prejudices and beliefs that could result hiring bias.

Our analyses revealed that applicants often use Internet-generated resume templates that provide only a broad overview of candidate qualifications and lack consideration of discernable skills and competencies. Frequently, candidates infuse terms from the job description into the resume without evidence to support an understanding of or achievement of the required skill or competency. Providing more details in posting an available position, based on well-recognized research competencies and project-specific needs, will result in an increased capacity to quickly match qualified candidates to the position.

Our research found that many applications are from individuals who are new to the clinical research enterprise, emphasizing the need to determine skills that can be transferred from these other positions to positions in clinical research. A definition of a transferrable skill is “*a specific set of skills that don’t belong to a particular niche, industry or job; they are general skills that can be transferred between jobs, departments and industries”* [7]. Widely accepted transferrable skills are communication, problem-solving, teamwork, organization, and time management skills; these skills alone are not sufficient for hire as a CRC. The importance of having experience in a direct patient care role/clinical setting provides familiarity with common medical terminology, a skill set similar to tasks required for an entry-level CRC job, and working with an interdisciplinary team of healthcare professionals. In addition to these skills, working in indirect clinical roles provides transferrable skills in patient scheduling, data collection and storage of information, and skill set development, that is, venipuncture, sample management, and shipping.

A limitation of this project is that it focused only on the initial step in the hiring process of research staff. After initial screening has been done by the HR recruiter, PIs/hiring managers need to be highly engaged in matching qualified candidates to the specific needs and focus of the research project. For example, several qualified candidates may be advanced for a particular position and the PI may choose the candidate with previous experience in a community of interest, or advanced knowledge of instruments and/or datasets being used in the project. These specific skill sets would not be identified in an initial screen by the HR recruiter. PIs and hiring managers can also rely on CRC standards that have been developed by professional organizations in making informed hiring decisions.

This study took place in an academic health center with approximately 400 CRCs employed at any given time and organized into 4 levels of skill CRC 1–4. This large number of positions and application facilitated the development of these screening guidelines. Smaller organizations and non-academic settings may require more dependence on recruiting CRCs with no previous research or healthcare experience. In those situations, transferrable skills are critical and may require more adaptability of the candidates. This may be a particular challenge in assessing individuals who have just graduated from undergraduate programs and may have limited transferable skills. The willingness of the PI to train employees in new skills may influence the hiring decision. We found delineating transferrable skills for the entry-level CRC facilitated eliminating non-qualified candidates from consideration, candidates that would likely require extensive onboarding leading to delayed project start-up. Institutions have developed unpaid research rotations and/or internships for students, that can facilitate their potential hiring after graduation.

Within many institutions, advancement in the CRC role is dictated by longevity in a research position. Hiring into the correct job level has implications for retention. If advancement requires 1–2 years of experience, an employee may choose to change jobs and leave the institution if they can advance to a higher level and increase their salary. Leaving the institution results in the loss of institutional knowledge and experience and adds to the cost of having to recruit and train a new employee. From the employee’s perspective, the cost difference on average for hiring between a CRC 1 and CRC 2 position; or CRC 3 and CRC 4 position is between $5200–6600/year. One HR administrator placed the cost to replace a CRC at $50–60K based on recruitment and hiring costs, employee orientation, and time to bring the new employee up to speed on a project.

This project emphasizes the importance relationship between the PI/hiring officer posting a position and the HR recruiter. Unfortunately, PIs may post CRC positions specifying level and salary based on the budget of the work and funds available instead of the expertise that is needed on the project. If the research project is underfunded and limited to hiring one employee, selecting an underqualified candidate at a lower CRC level of experience may jeopardize the project meeting critical milestones. One HR recruiter remarked on reviewing a PI’s list of job requirements for a CRC 1 position, “champagne taste on a beer budget.” Frustration with the mismatch of project needs, employee skill set, and PI expectations are recognized to increase job dissatisfaction and affect retention [8]. The HR recruiter can play an important role in supporting new PIs in understanding the range of CRC skills and the individuals that the project budget can afford. Many PIs are hiring research staff for the first time and these projects require substantial skills and experiences. Hiring the right candidate for the CRC position is only the first step. New PIs also need support for ongoing staff training and management with the ultimate goal of retaining staff [9].

The literature is mixed regarding hiring an overqualified applicant for a position [10]. Some recruiters and PI/hiring managers believe an overqualified candidate will quickly become bored and dissatisfied with job wages, responsibilities, and career advancement, and leave the position after a short time [11]. In a tight job market, overqualified applicants may take a lower-level position to gain entry into a system or use the position as a stepping stone within an institution to other positions. Motivation of the applicant for taking a job lower than their qualification status is a key factor that should not be dismissed when selecting to interview. Maltarich, Nyberg, and Reilly posit the relationship between cognitive ability and voluntary turnover is dependent on the cognitive demands of the job [12]. Hariri *et al*. identified a positive relationship with creative performance in the overqualified employee citing contextual factors such as wanting to work with a specific mentor or work on an intriguing new project [13]. For these employees, creating a suitable environment is key to job satisfaction [14]. It is important to remember, resume review using the hiring guidelines provides only an initial screening, reducing the number of candidates who may be underqualified or unsuitable for the job. The interview provides the opportunity to evaluate a candidate’s fit with the job. The role of HR is to provide top candidates to the PI for consideration. The human interaction component cannot be totally removed from hiring the candidate whose talent “best matches” the needs of the project.

## Conclusion

This project highlighted the large number of individuals who are interested in obtaining positions on clinical research projects. To recruit the most qualified individuals, investigators should view HR recruiters as partners and develop accurate resume screening methods to improve the hiring process. Regardless of the size of an organization’s research enterprise guidelines, screening guidelines for required skills and qualifications can be developed. We successfully developed guidelines for categorizing CRC applicant resumes from entry level to advanced position with the aim of improving the ability of HR to eliminate non-qualified candidates from the applicant pool. Key factors that should be included in the screening process include experience in direct/indirect clinical settings and roles, defined transferrable skills, academic degree focus, level of education, and clinical research experience. Foreign-trained PhD and MD candidates along with laboratory/bench researchers and new graduates need special consideration. Developing structured guidelines for HR recruiter use will reduce bias and improve the matching of applicant resumes to different levels of CRC jobs and can lead to improved selection of top candidates to advance to interview. Improved applicant to job matching offers an advantage to reduce hiring time, anticipate training needs, and shorten the timeline to active project engagement. While this project took place in a large academic setting, most organizations have recruiters in human resources who work to post positions, screen applicants, and sometimes receive references. Taking the time to know HR recruiters and view them as partners in the hiring process will result in overall process improvement.

## Supplementary material

For supplementary material accompanying this paper visit https://doi.org/10.1017/cts.2021.853. click here to view supplementary material
