# Supplementary material for: Finding the right candidate: Developing hiring guidelines for screening applicants for clinical research coordinator positions
Source: J Clin Transl Sci. 2021 Sep 22;6(1):e20. doi: 10.1017/cts.2021.853 (PMC8889228; doi:10.1017/cts.2021.853)
Supplement: Supplementary file 1 [file ctssup.zip › S2059866121008530sup001.docx]

**Supplementary Materials**

Table 1 Roles & Responsibilities of CRCs by Level

| **CRC I (entry level)** |
| --- |
| - Responsible for administrative activities associated with clinical trial conduct - Completes source documents/case report forms and performs data entry - Maintains and stores research data - Assists with participant scheduling |
| **CRC II (entry level)** |
| - Manages research project databases, developments study related documents, and completes source documents/case report forms - Interfaces with research participants and study sponsors - Determines eligibility and consents study participants according to protocol |
| **CRC III (intermediate level)** |
| - Independently manages significant and key aspects of a large clinical trial or all aspects of one or more small trials, or research projects - Trains and provides guidance to less experienced staff - Interfaces with research participants and resolves protocol issues - Interacts with study sponsors and monitors and reports SAEs - Resolves study queries - Provides leadership in determining, recommending, and implementing improvements to policies and procedures - Monitors IRB submissions |
| **CRC IV (advanced level)** |
| - Functions as a team lead to recruit, orient, and supervise research staff - Independently manages the most complex research administration activities - Determines effective strategies for promoting/recruiting/retaining research participants - Responds to requests and questions throughout the study life cycle |
