# Supplementary material for: Finding the right candidate: Developing hiring guidelines for screening applicants for clinical research coordinator positions
Source: J Clin Transl Sci. 2021 Sep 22;6(1):e20. doi: 10.1017/cts.2021.853 (PMC8889228; doi:10.1017/cts.2021.853)
Supplement: Supplementary file 1 [file ctssup.zip › S2059866121008530sup002.docx]

**Supplementary Materials**

Table 2 Final CRC Hiring Guidelines

| **CRC I** |
| --- |
| 1. High School Diploma, GED or Program Certificate (CNA, MA, Phlebotomy, Lab Tech) AND 1-year experience in a clinical setting/clinical role. 2. Associate Degree or 2 years of college AND 1 year experience in a clinical setting/clinical role. 3. Bachelor’s Degree or Master’s Degree in a scientific or health related field. 4. Bachelor’s AND Master’s Degree in a non-scientific field or health related field AND 1-year experience in a clinical setting/clinical role. |
| **CRC II** |
| 1. High School Diploma, GED or Program Certificate (CNA, MA, Phlebotomy, Lab Tech) AND 1-year experience in a clinical setting/clinical role. 2. Associate Degree or 2 years of college with 1 year experience in a clinical setting/clinical role AND 1 year of clinical research experience. 3. Bachelor’s Degree or Master’s Degree in a scientific or health related field AND 1 year of clinical research experience. 4. Master’s of Clinical Research (MSc); no clinical research required. 5. MD or PhD in a scientific or health related field (Includes unlicensed US, foreign trained MD’s). 6. PhD in a non-scientific or non-health related field AND 1 year in a clinical setting/clinical role; no clinical research required. |
| **CRC III** |
| 1. High School Diploma, GED or Program Certificate (CNA, MA, Phlebotomy, Lab Tech) AND 3 years of experience in clinical research. 2. Technical Diploma (LPN, Medical Assistant, Associate Degree or 2 years of college AND 3 years of experience of clinical research. 3. Bachelor’s or Master’s Degree any field AND 2 years of clinical research experience. 4. Master’s of Clinical Research (MSc) AND 1 year of clinical research experience. 5. MD or PhD in a scientific or health related field (Includes unlicensed US, foreign trained MD’s) AND 1 year of clinical research experience. 6. PhD in a non-scientific or non-health related field AND 2 years of clinical research experience. 7. Laboratory Researcher: Laboratory research required AND 1 year of clinical research experience.    1. Bachelor’s in scientific field AND 5 years’ research lab experience.    2. Master’s in scientific field AND 3 years’ research lab experience. |
| **CRC IV** **Must be a Certified Clinical Research Coordinator* |
| 1. High School Diploma, GED or Program Certificate (CNA, MA, Phlebotomy, Lab Tech) AND 5 years of experience in clinical research. 2. Technical Diploma (LPN, Medical Assistant, Associate Degree or 2 years of college AND 5 years of experience of clinical research. 3. Bachelor’s Degree AND 4 years of clinical research experience 4. Master's degree AND 3 years of clinical research experience. 5. Master’s of Clinical Research (MSc) AND 2 years of clinical research experience. 6. MD or PhD in a scientific or health related field (Includes unlicensed US, foreign trained MD’s) AND 2 years of clinical research experience. 7. PhD in a non-scientific or non-health related field AND 3 years of clinical research experience. 8. Laboratory Researcher: Laboratory research required AND 1 year of clinical research experience    1. Bachelor’s in scientific field AND 6 years’ research lab experience    2. Master’s in scientific field AND 4 years’ research lab experience. |
